# What Is Temperature? Modern Outlook on the Concept of Temperature

**DOI:** 10.3390/e22121366

**Published:** 2020-12-03

**Authors:** Edward Bormashenko

**Affiliations:** Chemical Engineering Department, Engineering Faculty, Ariel University, P.O. Box 3, Ariel 407000, Israel; edward@ariel.ac.il

**Keywords:** temperature, quantum Carnot engine, relativistic Carnot cycle, metrics of the configurational space, Landauer’s principle, entropic force

## Abstract

The meaning and evolution of the notion of “temperature” (which is a key concept for the condensed and gaseous matter theories) are addressed from different points of view. The concept of temperature has turned out to be much more fundamental than conventionally thought. In particular, the temperature may be introduced for systems built of a “small” number of particles and particles at rest. The Kelvin temperature scale may be introduced into quantum and relativistic physics due to the fact that the efficiency of the quantum and relativistic Carnot cycles coincides with that of the classical one. The relation of temperature with the metrics of the configurational space describing the behavior of systems built from non-interacting particles is demonstrated. The role of temperature in constituting inertia and gravity forces treated as entropy forces is addressed. The Landauer principle asserts that the temperature of a system is the only physical value defining the energy cost of the isothermal erasure of a single bit of information. The fundamental role of the temperature of the cosmic microwave background in modern cosmology is discussed. The range of problems and controversies related to the negative absolute temperature is treated.

## 1. Introduction

What is temperature? Intuitively, the notions of “cold” and “hot” precede the scientific terms “heat” and “temperature.” Carus noted in *De rerum natura* that “warmth” and “cold” are invisible and this makes these concepts difficult to understand [1]. The scientific study of heat started with the invention of the thermometer [2]. The operational definition of temperature is shaped as follows: Temperature is what we measure with a thermometer [3]. Galileo and his contemporaries were already using thermometers around 1600. Robert Boyle, Robert Hooke, and Edmond Halley suggested to use the standard “fixed points,” namely phenomena that could be used as thermometric benchmarks because they are known to always take place at the same temperature. Jean-Andre de Luc and Henry Cavendish made much research in order to establish these fixed points [2]. It turned out that an accurate establishment of these points poses as extremely difficult experimental problems. Consider, for example, the temperature of water boiling. We have to answer exactly and experimentally what “water boiling” is. Try to boil water and you will recognize that it is a complicated process, divided temporally into common boiling, hissing, bumping, explosion, and bubbling. Now, which of these is true boiling? The detective story of the development of accurate thermometers is excellently reviewed in the monograph “Inventing Temperature. Measurement and Scientific Progress” [2].

The theoretical breakthrough in the understanding of temperature occurred when Carnot discovered the temperature function, which was gradually developed over a period of 30 years by Clapeyron, Helmholtz, Joule, Rankine, Thomson (Kelvin), and Clausius [4,5]. In Thomson’s final resolution of the problem, Carnot’s function simply determined the “absolute” thermodynamic temperature scale [4]. Indeed, the efficiency of the Carnot engine $\eta =1-\frac{T_{2}}{T_{1}}$ is independent of any specific material constants and depends on the absolute temperatures of the hot $T_{1}$ and cold $T_{2}$ baths only [5,6].

Let us quote William Thomson: “The relation between motive power and heat, as established by Carnot, is such that quantities of heat, and intervals of temperature, are involved as the sole elements in the expression for the amount of mechanical effect to be obtained through the agency of heat; and since we have, independently, a definite system for the measurement of quantities of heat, we are thus furnished with a measure for intervals according to which absolute differences of temperature may be estimated [2].”

It should be emphasized that the efficiency of the Carnot engine is independent of the number of particles constituting the working fluid of the engine [7]. This makes possible the construction of the absolute temperature scale, suggested by Kelvin, for small-scale physical systems. The nontrivial problem of the matching of thermometric and absolute (Kelvin) temperature scales is discussed in detail in [6].

Remarkably, the efficiency of the Carnot cycle remains the same for a quantum mechanical cycle exploiting a single quantum mechanical particle confined to a potential well [8]. The efficiency of this engine is shown to be equal to the well-known Carnot efficiency, because quantum dynamics is reversible [8]. Moreover, the efficiency of the Carnot cycle remains the same for the relativistic Carnot engine. The relativistic transformation of temperatures remains a subtle and open theme, in which different expressions for this transformation have been suggested [9,10].

Planck and Einstein suggested that the relativistic transformation of temperatures is governed by: $T=T_{0\;}\sqrt{1-\frac{u^{2}}{c^{2}}}=\frac{T_{0}}{\gamma }$$;\;\gamma =\frac{1}{\sqrt{1-\frac{u^{2}}{c^{2}}}}$ where $T_{0}\;\mathrm{is}$ Kelvin’s temperature as measured in the rest system of coordinates and *T* is the corresponding temperature detected in the moving system [9]. By contrast, Ott suggested for the same transformation $T=\gamma T_{0}\;$ [10]. It was also suggested that the universal relativistic transformation for temperature does not exist [11,12,13].

However, it is easily seen that the efficiency of the Carnot cycle remains the same for the linear relativistic transformations of temperature shaped as: $T=\alpha T_{0};\;\alpha =const$, whatever the value of the constant. Thus, we recognize that the efficiency of the Carnot engine demonstrates remarkable stability and insensitivity to the make-up of the engine, number of particles constituting the working fluid of the engine [8], quantum behavior of the particles, and also to the motion of frameworks. This fact enables the introduction of the Kelvin thermodynamic temperature scale in the realms of relativity and quantum mechanics. Somewhat surprisingly, the Carnot cycle and absolute temperature scale represent the interception point for classical, quantum, and relativistic physics.

## 2. Results and Discussion

### 2.1. Temperature as an Average of the Kinetic Energy and the Metrics of Configurational Space

The ubiquitous understanding of temperature is that the temperature of a substance is related to the average kinetic energy of the particles of that substance [14]. It seems that this idea belongs to Daniel Bernoulli [2]. We will demonstrate that this is a very narrow definition of temperature, and it does not always work. However, we start from this understanding of temperature and we will show that such an interpretation leads to the nontrivial relation of temperature to the metrics of the physical configurational space. Indeed, geometry enters into the realm of physics in its relation to the inertial properties of masses, in other words, in its relation to their kinetic energies [15]. Consider the system of *N* noninteracting point masses $m_{i}$. Their kinetic energy $E_{k}$ equals:

(1) $$E_{k}=\frac{1}{2}\overset{N}{\underset{i=1}{\sum }}\frac{1}{2}m_{i}v_{i}^{2}$$

Let us define an element ds of the 3*N* configurational space according to Equation (2):

(2) $$ds^{2}=2E_{k}dt^{2}=dt^{2}\overset{N}{\underset{i=1}{\sum }}m_{i}v_{i}^{2}=\overset{N}{\underset{i=1}{\sum }}m_{i}\left(dx_{i}^{2}+dy_{i}^{2}+dz_{i}^{2}\right),$$

Thus, the kinetic energy $E_{k}$ may be rewritten as:

(3) $$E_{k}=\frac{1}{2}m{\left(\frac{ds}{dt}\right)}^{2},$$

where $m_{i}=m=1.$ In this Euclidian configurational space, $\sqrt{m}x_{i};\sqrt{m}y_{i};\sqrt{m}z_{i}$ are the Cartesian coordinates. In this space, the kinetic energy of the system is represented by the kinetic energy of a single point mass with m=1. When $E_{k}=const$ takes place, this point moves with the constant velocity:

(4) $$\frac{ds}{dt}=\sqrt{\frac{2E_{k}}{m}}$$

Now, assume that our mechanical system, built of *N* noninteracting point masses $m_{i}$, is in thermal equilibrium with a heat bath at a fixed temperature *T* (from a statistical point of view, this means that the system is described by the canonical ensemble [16]). In this case, the averaged element of the configurational space is defined as follows:

(5) $$\bar{ds^{2}}=2dt^{2}\overset{N}{\underset{i=1}{\sum }}\frac{1}{2}m_{i}\bar{v_{i}^{2}}$$

We assume that the system is ergodic and demonstrates the same statistical behavior averaged over time as over the system’s entire possible state space; thus, the averaging in Equation (5) may be the time or ensemble averaging [16,17]. Thus, the temperature of the system may be introduced according to Equation (6):

(6) $$\frac{1}{2}m_{i}\bar{v_{i}^{2}}=\frac{3}{2}k_{B}T,$$

where $k_{B}$ is the Boltzmann constant. Hence, Equation (5) may be rewritten as:

(7) $$\bar{ds^{2}}=3Nk_{B}Tdt^{2}$$

and the velocity $v^{*}$ may be introduced:

(8) $$v^{*}=\sqrt{\frac{\bar{ds^{2}}}{dt^{2}}}=\sqrt{3Nk_{B}T}$$

(again, m=1 is assumed).

When the temperature of the system *T* is constant, its time evolution may be represented by the motion of a single point in the configurational 3*N*-space ($\bar{x_{i}}=\sqrt{\bar{v_{i}^{2}}}t$; $\bar{y_{i}}=\sqrt{\bar{v_{i}^{2}}}t$; $\bar{z_{i}}=\sqrt{\bar{v_{i}^{2}}}t$) with the constant velocity $v^{*}=\sqrt{3Nk_{B}T}=const.$ Thus, the metrics of the configurational space is completely defined by the temperature of the system. This conclusion is trivial; however, it is not obvious, due to the fact the kinetic energy of the system now is not constant, but fluctuates around the average value with a probability given by the Gibbs formula. Actually, Equation (8) reflects the well-known input, stating that the canonical ensemble does not evolve with time [16]. Hence, the temperature of the system in thermal equilibrium with the thermal bath defines the constant velocity of the single point (see Equation (8)), describing the motion of this point in the 3*N* configurational space, where coordinates are defined as follows: $\bar{x_{i}}=\sqrt{\bar{v_{i}^{2}}}t$; $\bar{y_{i}}=\sqrt{\bar{v_{i}^{2}}}t$; $\bar{z_{i}}=\sqrt{\bar{v_{i}^{2}}}t.$

### 2.2. Temperature, Energy, and Entropy: An Alternative Glance on the Temperature

An alternative look on the temperature emerges from the concept of entropy. Alternatively, the notion of “temperature” is introduced according to Equation (9):

(9) $$\frac{1}{T}={\left(\frac{\partial S}{\partial E}\right)}_{N}$$

where *S* and *E* are the energy and entropy of the system correspondingly and *N* is the number of particles constituting the system [3,5,16,18,19,20]. The inverse temperature, defined according to Equation (9), is seen as the rate of change in entropy of the system taking place with the change in its energy. In other words, the inverse temperature appears as a measure of energy necessary for the ordering of the system, estimated by its entropy, which in turn, is given by the Boltzmann formula $S=k_{B}lnW$, where *W* is the number of micro-states corresponding to a certain macro-state of a system [16]. Actually, this definition is very different from that supplied by Equation (6) relating the temperature to the averaged kinetic energy of particles. First, it does not imply averaging and may be introduced for the system built of an arbitrary number of particles. Indeed, the notions of entropy and energy may be introduced for the physical systems containing any number of particles, however small or large, and even for single-particle systems [21,22]. Second, it does not arise from the kinetic energy (motion) of particles. As a matter of fact, it may be successfully applied for systems of particles in rest, such as an ensemble of spins (elementary magnets) embedded into the magnetic field [18,20]. Thus, Equation (9) supplies a much more general definition of the temperature than that relating the concept of the temperature to the averaged kinetic energy of the system. Consider that the Kelvin definition of temperature, emerging from the Carnot cycle, also does not relate the temperature to the molecular motion. The substitution of Equation (9) into Equation (7) yields the nontrivial equation defining the average metrics of the configurational space:

(10) $${\left(\frac{\partial S}{\partial E}\right)}_{N}\bar{ds^{2}}=3Nk_{B}dt^{2}$$

and the velocity of the representing point in the configurational space:

(11) $$v^{*}=\sqrt{\frac{\bar{ds^{2}}}{dt^{2}}}=\sqrt{3Nk_{B}{\left(\frac{\partial S}{\partial E}\right)}_{N}^{-1}}$$

### 2.3. Entropy Forces and Fundamental Role of Temperature

The fundamental role of temperature becomes even more evident when we talk about the so-called entropic forces. An entropic force results from the entire system’s statistical tendency to increase its entropy, rather than from a particular underlying force on the atomic scale. The force equation is expressed in terms of entropy differences, and is independent of the details of the microscopic dynamics. In particular, there is no fundamental field associated with an entropic force. Consider the uniaxial isothermal compression of an ideal gas. This compression demands a force:

(12) $$f=-T\frac{dS}{dx}$$

where *T* and *S* are the temperature entropy of the gas [23]. Molecules constituting an ideal gas do not interact; thus, the energy of the ideal gas under isothermic compression remains the same; hence, the force *f* emerges only from the tendency of the gas to increase its entropy [23]. Thus, for a given ideal gas, its absolute temperature appears as a sole macroscopic parameter defining an entropic elasticity of the gas column. A similar purely entropic force is necessary to hold an ideal polymer chain separated by the end-to-end distance $\vec{R}$:

(13) $$\vec{f}=\frac{3k_{B}T}{Nb^{2}}\vec{R},$$

where *N* and *b* are the number and length of the Kuhn segment, respectively [24]. Again, for a given ideal polymer chain, temperature is a sole macroscopic parameter defining the entropic spring constant. Verlinde suggested recently that the gravitational force and inertia are also entropic forces [25]. This idea (to be discussed below in more detail) strengthens even more the significance of the notion of temperature.

### 2.4. The Landauer Principle and Informational Interpretation of the Temperature

The Landauer principle establishing the physical equivalent of information supplies an additional outlook on the temperature. In its simplest meaning, the Landauer principle states that the erasure of one bit of information requires a minimum energy cost equal to $k_{B}Tln2$, where *T* is the temperature of a thermal reservoir used in the process [26,27]. Landauer also applied the suggested principle to the transmission of information and reshaped it as follows: An amount of energy equal to $k_{B}Tln2$ (where $k_{B}T$ is the thermal noise per unit bandwidth) is needed to transmit a bit of information, and more if quantized channels are used with photon energies $h\nu >k_{B}T$ [28]. Actually, the Landauer principle converts the information into a physical value; Landauer himself stated that the “information is physical” [26]. The precise meaning, universality, evaluation, and interpretation of the Landauer principle were recently subjected to intensive and sometimes stormy scientific discussion [29,30,31,32,33,34,35,36,37,38,39]. Whatever the precise meaning of the Landauer Principle, it contributes to the re-construction of the fundamentals of physics on the informational basis, suggested by Wheeler in [40] and developed in [41,42,43,44]. It immediately follows from the Landauer Principle that the temperature of the system is the only physical value defining the energy cost of isothermal erasure of a single bit of information [26,27,28,37,38]. Again, recall that the temperature defined with Equation (9) is introduced not only for large Avogadro-number-scale systems but also for systems built of an arbitrary number of particles [21,22], and even for the single-particle systems, as it is well-illustrated by the minimal Szilard Engine, however classical [7,44,45], quantum [8,39], or relativistic. This means that the mass-equivalent of the single bit of information may be introduced, which is also completely defined by the temperature of the system [27,31,32,33,34,35,36,46]. The notion of temperature, seen from the perspective of the Landauer principle and Equation (9), is transformed into the fundamental physical quality, and it is not interpreted as “the averaged kinetic energy of particles,” as it is usually understood.

**Figure 1:**
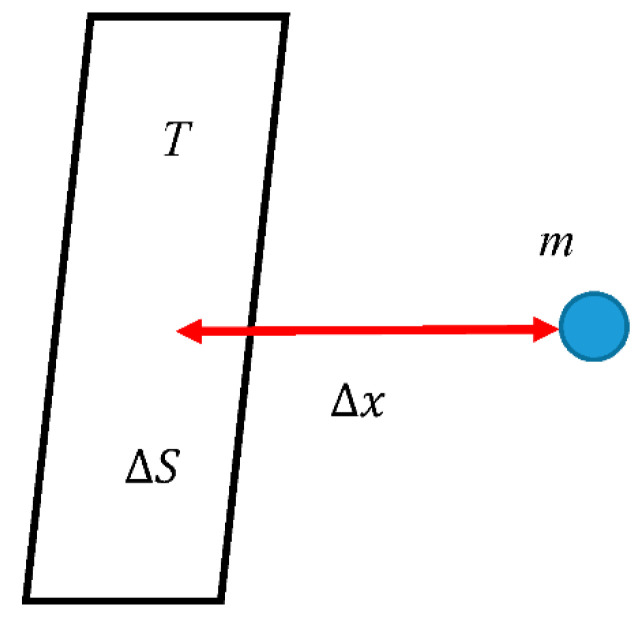
Origin of the inertia entropic force is illustrated [25]. A particle with mass *m* approaches a holographic screen possessing temperature *T*. ΔS is the entropy change near the screen.

Consider that the Landauer principle enables informational re-interpretation of mechanics [40,41,42,43,44]. When a particle is at rest or moves rectilinearly with constant speed, no information is transferred from one object to another. Indeed, for the transferring of at least one bit of information from a transmitter to a receiver, the particle (it may be a photon, electron, or a macroscopic particle) should be emitted and absorbed. Both of these processes necessarily demand the acceleration (deceleration) of a particle [44]. Let us estimate the minimal acceleration *a*, enabling the erasure of one bit of information. This acceleration emerges from the approach introduced in [25], in which the inertia force and gravity were treated as entropy forces, discussed in the previous section. This approach is based on the so-called holographic principle, assuming that the description of a volume of space can be thought of as encoded on a lower-dimensional boundary to the region (i.e., screen)—such as a gravitational horizon [47,48,49]. In the entropy/information-based approach suggested in [25,48,49], space-time is considered emerging phenomena. The authors considered a holographic screen, and a particle of mass *m* that approaches it from the side at which space time has already emerged, as depicted in Figure 1 [25]. It was adopted that the change in entropy near the screen is linear with displacement Δx:

(14) $$\Delta S=2\pi k_{B\;}\frac{mc}{\hbar }\Delta x$$

To understand why the change in entropy is proportional to the mass *m*, imagine splitting the particle into two or more lighter sub-particles. Each sub-particle then carries its own associated change in entropy after a shift Δx. As entropy and mass are both additive, it is natural to expect that the entropy change should be proportional to the mass. How does the inertia force arise? The author of [25] exploited the analogy with osmosis across a semi-permeable membrane. When a particle has an entropic reason to be on one side of the membrane and the membrane carries a temperature, it will experience an entropy force equal to |FΔx|=|TΔS|, which is already known to us from Equation (12). In [47], it was demonstrated that an observer in an accelerated frame experiences a temperature:

(15) $$T=\frac{1}{2\pi }\frac{\hbar a}{c}$$

Combining |FΔx|=|TΔS| and Equations (14) and (15) immediately yields for the inertia force |F|=|ma|. Thus, temperature plays a decisive role in the constituting of the inertia force, when treated as an entropic force. Combining Equation (15) with the Landauer principle yields Equation (16):

(16) $$a=\frac{2\pi ln2ck_{B}T}{\hbar },$$

which supplies the minimal acceleration necessary for erasing one bit of information at temperature *T*.

### 2.5. Fundamental Role of the Cosmic Background Temperature

The fundamental role of the notion of temperature becomes even more pronounced in the context of the effect of the cosmic microwave background [50]. The discovery and interpretation of the cosmic microwave background in 1965 by Arno Penzias, Robert Wilson, and Robert H. Dicke was a turning point in modern-century cosmology [50]. The discovery supported the now-well-established cosmological paradigm, broadly known as the Big Bang cosmology [51]. The cosmic microwave background (CMB) in the Big Bang cosmology is electromagnetic radiation as a remnant from an early stage of the universe, also known as “relic radiation.” The CMB has a thermal blackbody spectrum at a temperature of 2.73548 ± 0.00057 K. It appears that this temperature today serves as one of the most important physical constants. Probably the most significant and most frequently cited consequence of the standard hot Big Bang interpretation of the CMB is the limit the background temperature sets on the fraction of universal density that can be in the form of baryonic matter. The physical picture underlying this prediction is simple: The baryonic number is (at least approximately at timescales comparable to the Hubble time, neglecting effects of the hypothetical proton decay and other very slow processes) a conserved quantity, and the vast majority of photons currently existing in the universe are CMB photons, so the photon-to-baryon ratio today is essentially the same as it was at the time of decoupling, at redshift. Therefore, fixing the photon density per co-moving volume, coupled with limitations on the baryon-to-photon ratio in the early universe (provided by the theory of primordial nucleosynthesis [50,51,52,53]), gives a unique handle on the total cosmological baryon density.

### 2.6. Boltzmann and Gibbs Temperatures: Is a Negative Absolute Temperature Possible?

**Figure 2:**
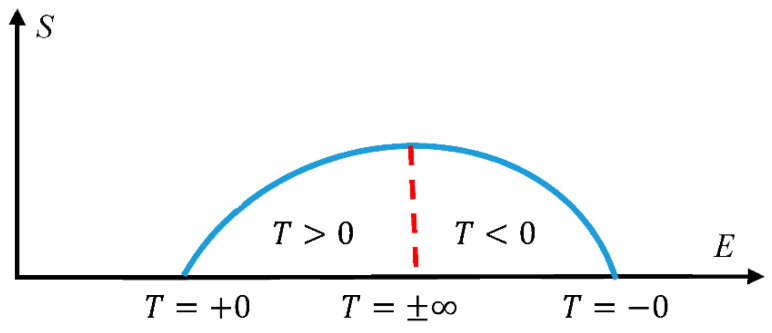
Origin of negative temperatures in population-inverted systems with a bounded spectrum is illustrated. The field of negative absolute temperatures is located above the infinite absolute temperatures [16,54,55].

The widespread understanding of the notion of temperature implies that an absolute Kelvin temperature is always positive. However, already in the classical textbook by Landau and Lifshitz, negative absolute temperatures are considered [16]. It was reported that negative absolute temperatures become possible in dielectric, paramagnetic materials, such as crystals of LiF in the population-inverted regime, when a spectrum of the system is bounded [16,19,54]. Consider the dependence of entropy of the physical system on its entropy S(E), depicted in Figure 2. Such a dependence, indeed, enables negative absolute temperatures, defined with Equation (9). The portion of the plot at which ${\left(\frac{\partial S}{\partial E}\right)}_{N}<0$ corresponds to negative absolute temperatures. It is noteworthy that the field of negative absolute temperatures according to the interpretation, suggested in [16], is located above the infinite below temperatures, and not below zero of the absolute (Kelvin) temperature scale. The concept of the negative absolute temperature was criticized recently in [55]. Obviously, negative absolute temperatures, when realized experimentally, enable a thermal (Carnot) engine with an efficiency larger than unity. The authors of [55] split the definition of the absolute temperature, supplied by Equation (9), into two, summarized by Equations (17) and (18):

(17) $$\frac{1}{T_{B}}={\left(\frac{\partial S_{B}}{\partial E}\right)}_{N}$$

(18) $$\frac{1}{T_{G}}={\left(\frac{\partial S_{G}}{\partial E}\right)}_{N},$$

where $T_{B}$ and $S_{B}$ are the Boltzmann temperature and entropy, respectively, given by Equations (17) and (19); $T_{G}$ and $S_{G}$ are the Gibbs temperature and entropy given by Equations (18) and (20), respectively.

(19) $$S_{B}=k_{B}ln\left(\epsilon \omega \right);\;\omega =tr\left[\delta (E-H)\right]$$

(20) $$S_{G}=k_{B}lnW$$

where ϵ is a constant with dimensions of energy, ω is the density of states, *W* is the number of microscopic configurations (micro-states), and *H* is the Hamiltonian of the system. The equation relating $T_{B}$ and $T_{G}$ was derived in ref. [55]:

(21) TB=TG1−kBC,

where $\;C=\frac{\partial E}{\partial T_{G}}$ is the total thermal capacity of the system associated with $T=T_{G}.$ As recognized from Equation (21), the difference between $T_{B}$ and $T_{G}$ becomes relevant if *C* is close to or smaller than $k_{B};$ in particular, $T_{B}$ is negative if $0<C<k_{B}$, as realized in the population-inverted regime [55]. It was demonstrated that no controversy exists, if the Gibbs definition of temperature expressed by Equations (18)–(20) is adopted [55]. In this case, absolute temperature remains positive even for systems with a bounded spectrum [55].

## 3. Conclusions

The universal absolute temperature scale suggested by Kelvin is possible due to the amazing robustness of the Carnot cycle. The efficiency of the Carnot engine is independent of the number of particles constituting the working fluid [7] and also of the specific material constants, depending on the temperatures of the hot $T_{1}$ and cold $T_{2}$ baths only. Moreover, it remains the same for the quantum [8] and relativistic Carnot engines. This fact allows the introduction of the Kelvin absolute thermodynamic temperature scale in the realms of relativity [9] and quantum mechanics [8]. This converts the notion of temperature into the key concept of the modern physics of condensed and gaseous matter [19]. The relation of the temperature to the metrics of the configurational space describing the behavior of the system built from non-interacting particles is treated. The temperature defined with the equation $\frac{1}{T}={\left(\frac{\partial S}{\partial E}\right)}_{N}$ (where *S* and *E* are the entropy and energy of the system correspondingly) is not related to the averaging of the kinetic motion of particles constituting the system, and may be introduced for systems containing an arbitrary number of particles, including those at rest [18,19,20].

From the point of view of the physics of information, the temperature of the system appears as the only physical value defining the energy cost of the erasure of a single bit of information. The crucial role of temperature for constituting entropic forces is treated. This role becomes even more significant when inertia force and gravity are treated as entropic forces [25]. The fundamental importance of the temperature of the cosmic microwave background in the grounding of basic ideas of modern cosmology is addressed [50]. The temperature of the CMB has turned out to be one of the most important fundamental physical constants limiting the value of the photon–baryon ratio in the Universe. The range of problems and controversies related to the notion of the negative absolute temperature is discussed (recall that negative temperatures were introduced for quantum population-inverted systems with a bounded spectrum [16,54]). Negative absolute temperatures enable the existence of controversial Carnot engines with an efficiency larger than unity [55]. The fine structure of the notion of temperature, split into the Boltzmann and Gibbs temperatures, is addressed [55]. No controversy exists if the Gibbs definition of temperature is adopted [55]. In this case, absolute temperature remains positive even for systems with a bounded spectrum [16,54,55].
